# Non-Technical Skill Assessment and Mental Load Evaluation in Robot-Assisted Minimally Invasive Surgery

**DOI:** 10.3390/s21082666

**Published:** 2021-04-10

**Authors:** Renáta Nagyné Elek, Tamás Haidegger

**Affiliations:** 1 Antal Bejczy Center for Intelligent Robotics, University Research and Innovation Center, Óbuda University, 1034 Budapest, Hungary; haidegger@irob.uni-obuda.hu; 2 Doctoral School of Applied Informatics and Applied Mathematics, Óbuda University, 1034 Budapest, Hungary; 3 John von Neumann Faculty of Informatics, Óbuda University, 1034 Budapest, Hungary; 4 Austrian Center for Medical Innovation and Technology, 2700 Wiener Neustadt, Austria

**Keywords:** non-technical skills, Robot-Assisted Minimally Invasive Surgery, skill assessment, surgical skills

## Abstract

**BACKGROUND:** Sensor technologies and data collection practices are changing and improving quality metrics across various domains. Surgical skill assessment in Robot-Assisted Minimally Invasive Surgery (RAMIS) is essential for training and quality assurance. The mental workload on the surgeon (such as time criticality, task complexity, distractions) and non-technical surgical skills (including situational awareness, decision making, stress resilience, communication, leadership) may directly influence the clinical outcome of the surgery. **METHODS:** A literature search in PubMed, Scopus and PsycNet databases was conducted for relevant scientific publications. The standard PRISMA method was followed to filter the search results, including non-technical skill assessment and mental/cognitive load and workload estimation in RAMIS. Publications related to traditional manual Minimally Invasive Surgery were excluded, and also the usability studies on the surgical tools were not assessed. **RESULTS:** 50 relevant publications were identified for non-technical skill assessment and mental load and workload estimation in the domain of RAMIS. The identified assessment techniques ranged from self-rating questionnaires and expert ratings to autonomous techniques, citing their most important benefits and disadvantages. **CONCLUSIONS:** Despite the systematic research, only a limited number of articles was found, indicating that non-technical skill and mental load assessment in RAMIS is not a well-studied area. Workload assessment and soft skill measurement do not constitute part of the regular clinical training and practice yet. Meanwhile, the importance of the research domain is clear based on the publicly available surgical error statistics. Questionnaires and expert-rating techniques are widely employed in traditional surgical skill assessment; nevertheless, recent technological development in sensors and Internet of Things-type devices show that skill assessment approaches in RAMIS can be much more profound employing automated solutions. Measurements and especially big data type analysis may introduce more objectivity and transparency to this critical domain as well. **SIGNIFICANCE:** Non-technical skill assessment and mental load evaluation in Robot-Assisted Minimally Invasive Surgery is not a well-studied area yet; while the importance of this domain from the clinical outcome’s point of view is clearly indicated by the available surgical error statistics.

## 1. Introduction

Minimally Invasive Surgery (MIS) induced a paradigm change in medicine; however, it presented new challenges for surgeons [1,2]. In the case of MIS—against traditional, open-access surgery—inside organs are reached through small skin incisions with laparoscopic instruments, and the operating area is visualized with an endoscopic camera. During MIS, the operator (surgeon) has to work in a team as a leader, he/she gives instructions to a camera handler assistant and the other operating room members, while he/she has to constantly monitor the operating area on a 2D screen in an uncomfortable position. Thus, despite the clear benefits of MIS, including the smaller scars and faster recovery time, there are drawbacks for the physicians, such as the limited motion space, complicated instrument control, not ergonomic environment and the two dimensional endoscopic camera image.

**Figure 1:**
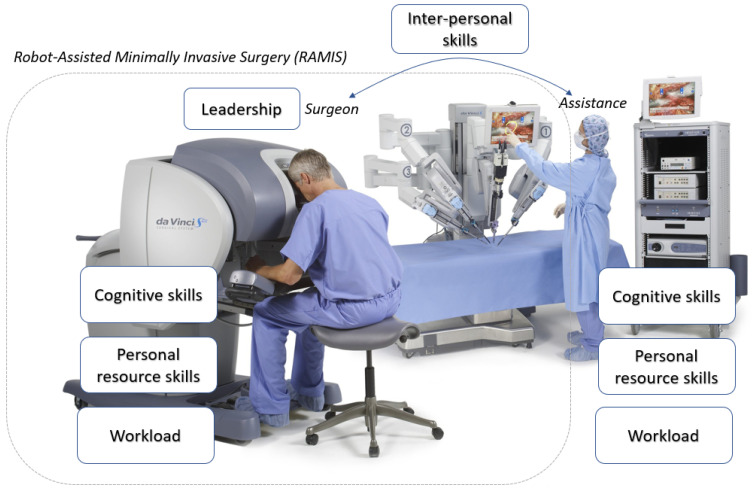
The da Vinci Surgical System with the identified non-technical skills and workload. The surgeon operates at the master side of the system, while the assistants can help them work at the patient side. The patient side arms are controlled by the surgeon with the master arms. Robot-Assisted Minimally Invasive Surgery requires not just technical skills, but non-technical skills as well from the operating crew, namely inter-personal skills, leadership, cognitive skills and personal resource skills, while they have to deal with the workload. Original image credit: Intuitive Surgical Inc. [10].

Robot-Assisted Minimally Invasive Surgery (RAMIS) was the next step in the evolution of MIS: it provided an improved vision system, more accurate and intuitive instrument control and an ergonomic master console [3,4,5]. The most successful RAMIS system is the da Vinci Surgical System (Intuitive Surgical Inc., Sunnyvale, CA), which is a teleoperated, master–slave type surgical robot (Figure 1). The basic concept of a remote-controlled telesurgical system was created at the National Aeronautics and Space Administration (NASA) in around 1971, originally planned to be used for remote surgeries, where the slave robot is on the spaceship. In the case of the da Vinci Surgical System, the surgeon sits at an ergonomic master console, where he can operate with intuitively-controlled master arms. The surgeon can use pedals for special surgical instruments, such as a clutch, and to control the endoscopic arm, thus camera handling is only in the hands of the surgeon. At the patient side of the da Vinci, there are the remotely controlled slave arms, which accomplish the interventions minimally invasively with a motion mechanism called “Remote Center of Motion” (RCM), which can guarantee patient safety. The assistant crew works at the patient side of the da Vinci, where they can help the surgeon and support the intervention, such as they can change the surgical instruments during the operation. At the slave side, there is a 3D endoscopic camera, in which images are visualized in the screens placed in the master console; thus, the surgeon can see a 3D image of the operating area. The motion of the surgeon can be re-scaled on the patient side of the da Vinci, which can provide more accurate motion. However, the original idea of remote surgery was to operate with long distances; for safety reasons, at the moment it is not part of the clinical practice. It is important to note, against the name ‘robot’, the da Vinci Surgical System does not perform any kind of automation or decision making, neither decision support, the only very low-level automation in the da Vinci is tremor and abrupt motion filtering. A steep learning curve has been identified with the da Vinci [5,6]. Thus, despite the fact that RAMIS can decrease the mental workload of the surgeon as shown through by studies, RAMIS remains a challenging operation to perform not just physically, but mentally as well, because of the constant communication, teamwork, leadership, decision making and workload conditions (Figure 1) [7,8,9].

The improvements of RAMIS can help the surgeon, however, RAMIS is still a hard task to master; continuous training and feedback about the performance is crucial. Furthermore, the skills of the surgeon directly influence the outcome of the surgery. In surgical skill assessment, the Dreyfus model is often introduced [11]. The Dreyfus model shows the evolution of the learning process, and it can describe the typical features of the expertise levels at the different learning phases, such as a novice usually can only follow simple instructions, but an expert can well react to previously unseen situations. The Dreyfus model was fitted to surgical skills as well [12]. Surgical skill assessment improves training and provides quality assurance; therefore, it has benefits for surgeons and patients. While surgical skill assessment is available during training (such as with RAMIS simulators [13,14]), it is not the part of the everyday clinical practice yet [15,16]. Technical skill assessment is a well-studied area not just in traditional MIS, but in RAMIS as well [17]. Technical skills in RAMIS are related to the basic skills of the surgeon (knowing the instruments, using the right tools, etc.), the control of the robot and MIS tools (bimanual dexterity, endoscopic camera handling, clutch handling, instruments kept in view, etc.) and tissue handling (force sensitivity). Nevertheless, non-technical skill assessment is less objective.

**Figure 2:**
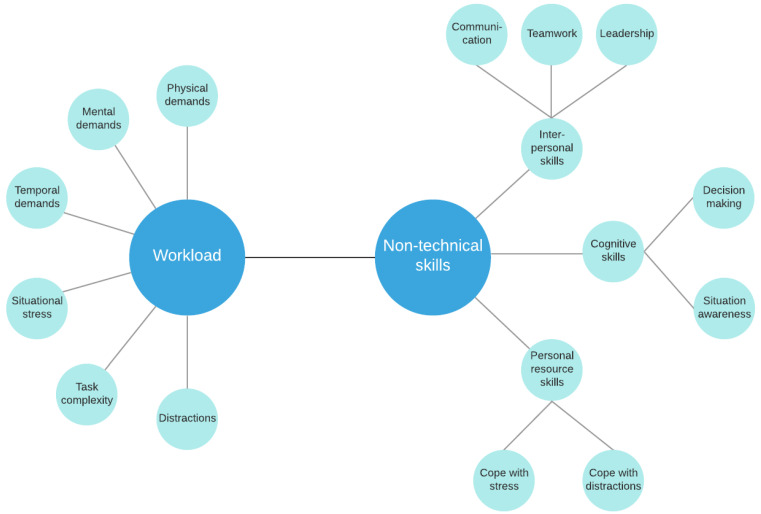
Workload categories and non-technical skills in Robot-Assisted Minimally Invasive Surgery (RAMIS), based on the SURG-TLX workload questionnaire and the ICARS expert-rating assessment tool. At the moment, there is no universal solution for mental workload assessment specifically created for RAMIS.

The workload on the surgeon—which represents the effort to perform a task—can be high in several domains of a procedure: there are mental, physical and temporal demands. Furthermore, task complexity (including multitasking, task novelty), situational stress and distractions can influence the outcome of the surgery [18,19] (Figure 2). Naturally, the same task can cause different workload to different operators. Non-technical skills related to the workload on the surgeon, furthermore, can directly affect surgical outcome [20]. Non-technical skills include communication, teamwork, task management, leadership, decision making, situational awareness and cope with stress, fatigue and distractions based on validated metrics, such as NOTSS and ICARS [21,22] (Figure 2). Situation Awareness (SA) has been recently investigated in other safety-critical domains, such as self-driving technologies, nevertheless, the SA assessment and quantification methods are very similar in both application areas [23]. While it is straightforward that technical skills are crucial for better surgical outcomes, non-technical surgical skills can be as important as technical skills. Clinical failures in the operating room may come from low non-technical skills of the surgeon rather than the lack of technical skills [24,25,26].

In the literature, three approaches for surgical performance assessment can be identified [17,27,28]:

- self-rating questionnaires,
- expert-based scoring and
- automated (sensor-based) skill assessment.

Questionnaires are filled out by the operator; thus, it is easy to implement and is subjective. Objective scoring is done by an expert panel, based on a standardized method [29]. Expert ratings are supposedly objective, but may be biased for personal reasons. Furthermore, they can be hard to implement, being human resource intensive. Automated skill assessment is based on objectively measurable parameters (such as applied forces, movement velocity, etc.), however, in most cases it is technically not easy to implement. Robotic surgical systems can provide a unique platform for objective skill assessment due to their built-in sensors providing a continuous flow of recordable kinematic and video data [3]. The original da Vinci Surgical System alone had 48 sensors. The mentioned surgical skill assessment approaches can be found in technical skill, non-technical skill and mental workload assessment as well. For mental workload assessment, questionnaires and automated solutions can be useful tools, and for non-technical skill assessment all of the methods (questionnaires, expert-rating and automated techniques) can be utilized.

The difference between traditional MIS and RAMIS mental workload was examined in some studies [30,31], demonstrating lower mental workload in the case of RAMIS. However, in these studies questionnaires created for traditional MIS were used, the main workload parameters in RAMIS are not yet defined. For RAMIS, non-technical skill assessment expert-rating methods originally created for traditional MIS can be found [32,33]. There is one metric specifically created for RAMIS non-technical expert-rating assessment (ICARS, [22]), which describes the most important non-technical skills in RAMIS (Figure 2). Non-technical skills are naturally hard to be measured automatically. The possibilities for automated RAMIS non-technical skill assessment are similar to traditional MIS, such as relying on physiological signals measured by additional sensors [34].

The goal of any kind of skill assessment is to employ automated and objective methods to measure the skills of the surgeon; thus avoiding biased assessment and the need for human resources. The built-in sensors of RAMIS can significantly ease automated skill assessment, since there are recordable kinematic and video parameters of the surgery (such as tool trajectory, orientation, velocity, etc.), which can provide input for skill assessment algorithms (statistical analysis or machine learning methods), towards traditional MIS, where these data are only available with additional sensors. Da Vinci Surgical System is a closed system; therefore, to analyze surgical data recorders is necessary, such as the da Vinci Research Kit (DVRK, developed by a consortium led by Johns Hopkins University and Worcester Polytechnic Institute), which can provide open-source hardware and software elements with complete read and write access to the first generation da Vinci arms [35].

**Figure 3:**
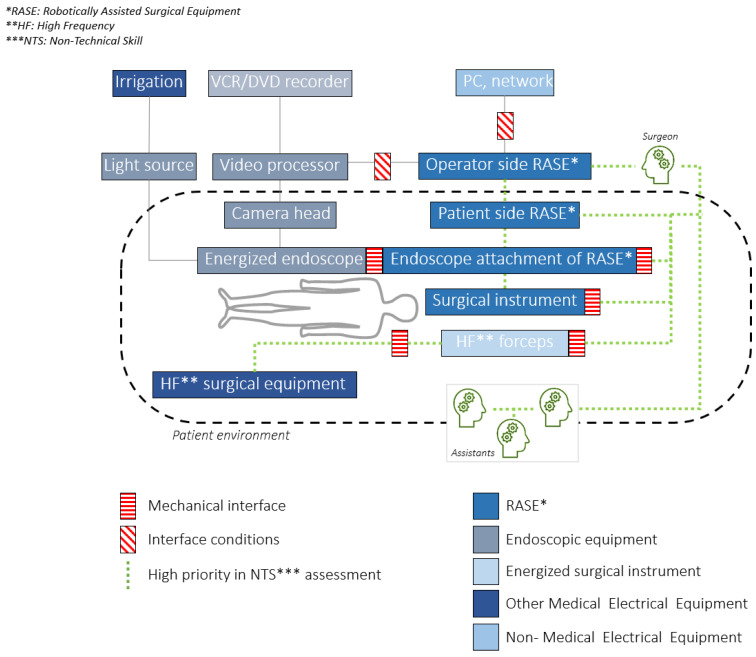
A Robot-Assisted Minimally Invasive Surgical system architecture and typical layout diagram with the most important sensor components in the case of non-technical skill assessment and mental load evaluation based on the International Electrotechnical Commission (IEC) 80601-2-77 robotic surgery safety standard [37].

To understand where non-technical skills can be identified in the case of RAMIS, high priority (interaction and communication) channels and interfaces have to be identified and analyzed. International Electrotechnical Commission (IEC) and International Organization for Standardization (ISO) published a new safety standard for surgical robots, the IEC 80601-2-77. In the standard, the components of RAMIS are defined, and a basic diagram of RAMIS is introduced [36,37]. Based on the proposed working diagram, we highlighted the most important components in non-technical skill assessment (Figure 3). For this, the following definitions were used from IEC 80601-2-77, following the taxonomy of the IEC 60601 medical device core standard:

- **Robotically Assisted Surgical Equipment—RASE**: ‘*Medical electrical equipment that incorporates programmable electrical medical system actuated mechanism intended to facilitate the placement or manipulation of a robotic surgical instrument*’ (the ISO 8373 standard strictly defines the term "robot" in the ISO domain, therefore the working group decided to use the more inclusive "Robotically Assisted" expression within RAMIS, while it is less commonly used in the domain).
- **Robotic surgical instrument**: ‘*Invasive device with applied part, intended to be manipulated by RASE to perform tasks in surgery*’.
- **High frequency (HF)**: ‘*less than 5 MHz and generally greater than 200 kHz*’.
- **HF surgical equipment**: ‘*medical electrical equipment which generates HF currents intended for the performance of surgical tasks, such as the cutting or coagulation of biological tissue by means of these HF currents*’.
- **Interface conditions**: *conditions that shall be fulfilled to achieve basic safety for any functional connection between RAMIS and other medical electrical equipment or non-medical electrical equipment in the robotic surgery configuration*.
- **Mechanical interface**: *mounting surface on RAMIS that allows for attachment of detachable accessories, components or parts that are mechanically manipulated by the RAMIS*.
- **Endoscopic equipment**: ‘*energized endoscope together with its supply unit(s), as required for its intended use*’ [36,37].

It is worth mentioning that the terminology of the ISO standard with respect to RASE slightly differs from RAMIS, mostly due to the fact that in ISO sense, the term “robot” is defined in a much narrower meaning.

In Figure 3 the components of RAMIS and the most important components in non-technical skill assessment are shown. Based on the literature findings, non-technical skill and workload can be assessed with the communication channel between the surgeon and the assistants, and with the cognitive and personal resource skills of the operating room crew, such as based on physiological signals or questionnaires, as it can be seen on the image, the surgeon’s decisions are inseparable from the control loop of RAMIS systems. It suggests that non-technical skills and workload might be shown in objectively measurable parameters, which means non-technical skill assessment is not necessarily different from technical skill assessment [38]. This may ease objective, automated non-technical surgical skill assessment in RAMIS. However, in the case of RAMIS, not many studies have examined this correlation.

In this paper, we review the recent results of non-technical skill and mental workload assessment in the case of Robot-Assisted Minimally Invasive Surgery. In the materials and methods section, we introduce the literature search strategy, following the standard PRISMA method. In Section 3.1, we show the mental workload assessment techniques in RAMIS (NASA-TLX, SURG-TLX, Multiple Resources Questionnaire, etc.). In Section 3.2, we show the expert rating techniques in RAMIS non-technical skill assessment. In Section 3.3, we overview the recent results in automated non-technical skill assessment techniques, furthermore, the possibilities and limitations of algorithm-based non-technical skill assessment. At the end of the paper, we review the relevant publications in a tabular form (Table 5), containing the following columns for easy comparability: reference, year of the publication, number of subjects involved, experimental environment, used assessment technique, measured non-technical skill, conclusion and quality of evidence. The paper ends with an appropriate discussion and conclusion.

## 2. Materials and Methods

To find relevant publications in the field of non-technical skill and mental workload assessment in RAMIS, the PubMed, Scopus and PsycNet databases were searched. The last search was performed in August 2020. To find relevant publications for mental workload assessment in RAMIS, we used the keywords ‘surgical robotics’ or ‘robotic surgery’ or ‘robot-assisted minimally invasive surgery’ and ‘workload assessment’ or ‘cognitive assessment’ or ‘NASA-TLX’ or ‘SURG-TLX’. In the case of expert rating and automated non-technical assessment, we use the keywords ‘surgical robotics’ or ‘robotic surgery’ or ‘robot-assisted surgery’ and ‘non-technical skill’ or ‘non-technical skill assessment’ or ‘NOTSS’ or ‘ICARS’. We included original articles about non-technical skills and mental workload assessment in RAMIS. We could not find any patents or software products matching the above criteria. We excluded publications that studied these assessment techniques in traditional MIS, not RAMIS, but included those which compared the two types of surgery with the non-technical skill assessment perspective. Due to the fact that we wanted to focus on RAMIS non-technical skills, we excluded publications about surgical process modeling, ergonomy (which considered physical workload only), technical skill assessment techniques, workflow assessments and reviews.

**Figure 4:**
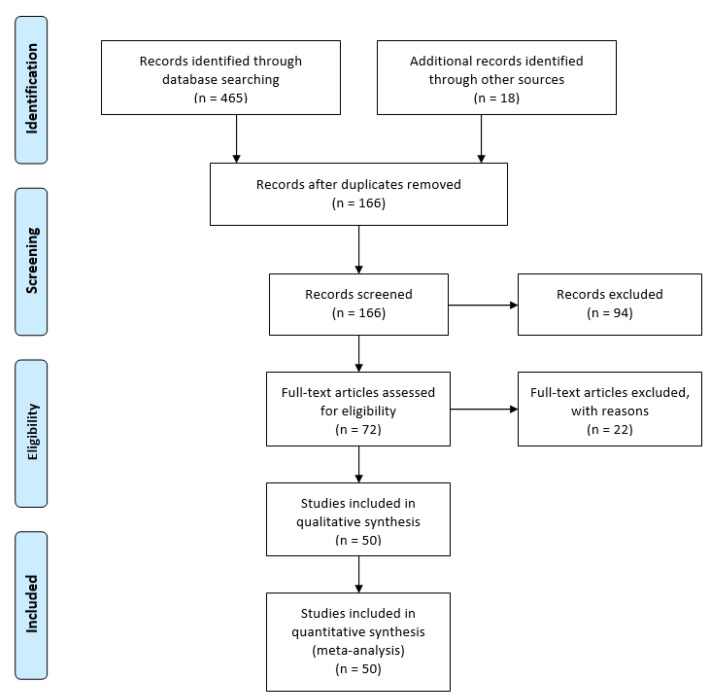
PRISMA chart of the literature search results.

**Figure 5:**
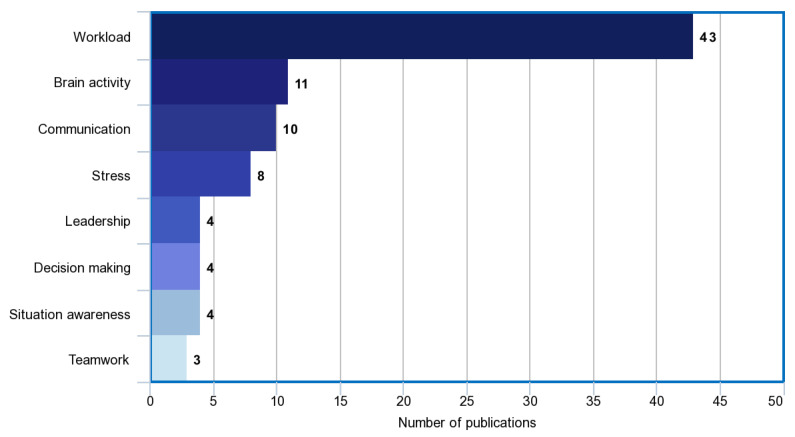
Bar chart of the literature search results. Each bar refers to the number of references identified and examined the particular feature/non-technical skill in RAMIS.

Fifty relevant publications were found in the field of non-technical skill and mental workload assessment in surgical robotics (Figure 4). From the relevant publications, the following research topics were identified: workload (42), brain activity (11), communication (9), stress (7), leadership (3), decision making (3), situation awareness (3) and teamwork (2) (Figure 5). The summarized results can be found in Table 5. We defined the quality of evidence based on the GRADE approach [39]. Study limitations, inconsistency of results, indirectness of evidence, imprecision and publication bias can decrease, and large magnitude of effect, plausible confounding and dose–response gradient can increase the quality of evidence in GRADE. To decide the quality of evidence, we carefully considered the impact of RAMIS workload and non-technical skill assessment research (which can increase or decrease the quality class). Based on the quality of evidence and the strength of recommendation, the following classes were defined:

- High: high-level of confidence in the effects;
- Moderate: confidence in the effects may change with future research findings;
- Low: confidence in the effects is very likely to change with future research findings;
- Very low: uncertainty about the effects.

## 3. Technical Approaches for Non-Technical Skill and Mental Workload Assessment in RAMIS

### 3.1. Mental Workload Assessment—Self-Rating Techniques

Performing a surgical procedure can be very stressful to the whole crew of the operating room. Fatigue (mental and physical) can naturally influence the outcome of the surgery; furthermore, time limits can cause serious stress and cognitive load on the surgeon, and working in a team can be disturbing in some cases. Workload is a term that represents the psychological cost to perform a task; it is human-specific, however, there are situations which can take a serious amount of mental workload from every operator. Workload can be defined with self-rating techniques, where a subject fills a questionnaire about his/her personal experience about the task workload. It is naturally a subjective technique, however, there are works in the literature which studied both subjective workload measurements and objective non-technical skill assessment metrics [32,40], or objective physiological parameters [30,34,41,42,43,44,45,46]. Workload measurements do not only help to assess the personal workload index, but also to define the main stressors and disturbing factors in surgery in general, furthermore, to provide personal training for novices as well.

NASA Task Load Index (NASA-TLX, created by NASA’s Ames Research Center in 1988) is a workload self-rate estimation metric, originally created for assessing workload in aviation [18,47]. NASA-TLX measures the workload on a subject with questions related to mental, physical and temporal demand, effort, performance and frustration level. The subject (which can be only one person or all team members) has to answer the questions on a 100-point-scale with 5-point steps (Table 1). NASA-TLX is a widely used technique for workload measurement in aviation, military and healthcare. NASA-TLX can be found in traditional MIS mental workload estimation [48,49,50,51,52], and employed in the case of surgical robotics workload assessment as well [8,32,34,40,41,43,45,53,54,55,56,57,58,59,60,61,62,63,64,65,66,67,68,69,70,71,72,73,74,75,76]. There are additional mental workload assessment techniques that are not originally created for surgery, and used in workload assessment for RAMIS. Such examples are:

- Multiple Resources Questionnaire (MRQ) [31,60,62,77,78].
- Dundee Stress State Questionnaire (DSSQ) [31,78,79].
- Rating Scale for Mental Effort (RSME) [42,80].
- Psychometric Testing of Interpersonal Communication Skills Questionnaire (PTICSQ) [81].
- Safety Attitudes Questionnaire (SAQ) [81,82]
- Wisconsin Card Sorting Test (WCST) [57,83].
- Coping Inventory of Task Stress (CITS) [31,84].
- Subjective Mental Effort Questionnaire (SMEQ) [85].
- Local Experienced Discomfort (LED) [85].
- Short Stress State Questionnaire (SSSQ) [62,86].

MRQ estimates workload with 17 items, and it is specifically useful for multitasking workload measurements [77]. SSSQ is based on DSSQ, and both target stress measurement [86], such as CITS [84]. RSME and SMEQ estimate mental effort on a 9-point scale from extreme effort to absolutely no effort. RSME is validated in healthcare as well [80]. LED examines physical discomfort during a task [85]. For team communication quality estimation PTICSQ was created [81]. SAQ was developed for healthcare, which examines employees’ satisfaction with the job, teamwork, management, safety, stress and working conditions [82]. WCST is a neuropsychological tool, which was originally created for cognitive strategy adaptation measurements [83].

Surgery Task Load Index (SURG-TLX) (created by the cooperation of the University of Hong Kong, University of Exeter and the Department of Urology, Royal Devon and Exeter Hospital in 2011) is a modified NASA-TLX metric for surgical workload measurements [87]. SURG-TLX estimates the workload based on mental demands, physical demands, temporal demands, task complexity, situational stress and distractions (Table 2, Figure 2). SURG-TLX was tested on the Fundamentals of Laparoscopic Surgery (FLS) peg transfer task under stress, such as fatigue, multitasking, distraction and task novelty. However, the metric was validated for surgery but we could only find a few RAMIS publications on this topic [42,44,54]. Nevertheless, this topic is well-studied in traditional MIS [88,89,90,91,92], and to the best of the authors’ knowledge there is no workload self-rating measurement metric specifically created for RAMIS.

Self-rating techniques are not resource-intensive to implement, and they do not require human support, however, they typically show a bias. After all, it is still necessary to consider the usage of self-rating techniques in automated or expert-rating focused NTS and workload assessment studies, because these questionnaires can provide an easy validation tool for correlation examinations. With self-rating tools, the real stressors of the surgery can be observed, and other approaches have to fit to the clinical relevance. Self-rating studies can be found in Table 5 under the following references: [30,31,32,34,40,41,42,43,44,50,56,57,59,60,61,62,63,64,66,68,69,70,71,72,75,76,81,85,93,94,95].

### 3.2. Non-Technical Skill Assessment—Expert Rating

In surgical skill assessment, expert rating techniques are widely used, not just in the case of technical skill assessment, but for non-technical skill assessment as well. Therein, an expert panel (usually 8–10 expert surgeons) assesses the skills of the practicing surgeon, based on a video recording of the procedure/training session, based on a validated set of requirements. Expert rating assessment is relatively easy to complete (compared to automated techniques), more objective than self-assessment, but it definitely requires significant human resources, and it can still be biased for personal reasons. At the moment, expert rating technique is the gold standard for automated skill assessment.

In the case of non-technical skill assessment, there are several different expert-rating metrics for traditional MIS, such as NOTECHS, OTAS and NOTSS (Table 3). A few publications were identified which studied NOTSS in the case of RAMIS [32,33,96]. For surgical robotics, there is one metric which specifically measures the non-technical skills of robotic surgeons [22]; the Interpersonal and Cognitive Assessment for Robotic Surgery (ICARS), developed by Raison et al. in 2017. It was created by 16 expert surgeons with the Delphi methodology [97]. In ICARS, there were 28 non-technical skills identified (Figure 2), in 3 main non-technical skill categories, namely interpersonal skills (communication/teamwork and leadership), cognitive skills (decision making and situational awareness) and personal resource skills (cope with stress and distractions, Table 4. However, we could only find one clinical study which used ICARS for non-technical surgical skill assessment [96]. Despite the disadvantages of expert-rating techniques (need for an expert surgeon’s input, time, bias), they can still be a more objective tool for automated technique validation. They can provide a model for NTS assessment through the critical NTS categories and the given points. Expert-rating studies can be found in Table 5 under the following references: [8,22,32,40,42,45,53,54,55,57,58,60,64,65,67,73,74,96,98,99].

### 3.3. Automated Non-Technical Skill and Mental Workload Assessment in RAMIS

Establishing the correlation between physiological signals, kinematic data or other objectively measurable features and non-technical skills or mental workload can lead to autonomous non-technical skill assessment in RAMIS.

A common approach to assess the non-technical skills of the surgeon is through the measurement of physiological signals. However, this has limitations: the physiological signals are often linked to a particular non-technical skill, such as stress level, but they do not show other important factors (situational awareness, teamwork, etc.). In the literature, we can find physiological measurements related to the stress level, such as:

- brain activity [103];
- skin temperature [104,105];
- nose temperature [106];
- heart rate [107];
- skin conductance [107];
- blood pressure [107];
- respiratory period [107];
- tremor [108];
- eye movement [109].

While these physiological signals are proven to be related to stress, they naturally have limitations in the usage of non-technical skills and cognitive load assessment. Such an example is skin conductance, which can be a useful technique to estimate workload [30], but it can be influenced by other physiological factors. Brain activity, heart rate and eye movement are the most studied signals in RAMIS, which can refer to more complex underlying behavior, such as technical skills [110], but the correlation between these signals and non-technical skills is harder to established.

In the literature, there are examples of the usage of an electroencephalogram (EEG) [34,43,45,69,70,72,93,111], given the fact that EEG measures the electrical activity of the brain [112]. While EEG is the most trivial physiological signal measurement technique for non-technical surgical skill assessment, the proven correlation between the measurable brain activity and non-technical skills is limited. Another approach for physiological signal-based mental workload assessment is the measurement of the heart rate (HR) [7,42,44,72,85]. However, the accuracy of HR measurements for cognitive load assessment was not enough in some cases, because there is no scale for maximum tolerated workload levels, and their related effects on the surgeon’s health [7]. The following forms of HR can be found in the non-technical skill assessment literature, however, the usage of them can be cumbersome [112,113]:

- simple HR;
- Heart Rate Variability (HRV);
- mean square of successive differences between consecutive heartbeats (MSSD);
- average heart rate (HRA).

Another objective method for non-technical skill or mental workload assessment is Functional Near-Infrared Spectroscopy (fNIRS) [44,114,115]. FNIRS is a functional neuroimaging technique to track the brain activity by monitoring the blood flow in the prefrontal lobe [116]. FNIRS shows a strong correlation with PET and fMRI data, yet it has better temporal resolution than fMRI but is limited compared to EEG; spatial resolution is more limited compared to fMRI, but better compared to EEG [117,118]. Furthermore, time of isovolumetric contraction (PEP) [119], electromyography and electrodermal [72] can also be used in mental workload assessment [85]; however, these signals can be influenced by the surgeon’s general health. Pupillary response is also studied in workload assessment [46].

As a summary, the following sensors/imaging techniques were studied in NTS and workload assessment in RAMIS (detailed in Table 5):

- magnetic pose trackers;
- EEG;
- ECG;
- fNIRS;
- skin conductance sensor;
- electromyograph (EMG);
- eye-gaze tracker;
- nose temperature and dryness sensor;
- heart rate monitor.

Adequate sensor solutions in RAMIS do not only constitute external ones, but there are built-in internal sensors as well, which can greatly facilitate NTS skill assessment (see Section 4 for future works) and have become proven tools for technical skill assessment in RAMIS:

- position sensors (encoders);
- gyroscopes;
- 2D/3D endoscopic camera.

In RAMIS research, there are typically integrated/employed sensors which are not directly related to NTS and workload assessment, but in most of the cases, their modalities show correlation with technical skills [17]. These sensor types include, but are not limited to, the following devices [120,121,122,123]:

- force sensors (strain gauges, capacitive sensors, piezoelectric sensors, optical sensors);
- tool position sensing (optical, electromagnetic);
- master/surgeon arm position sensing (external);
- wearable eyeglasses (Oculus Rift, Google Glass);
- tool thermal sensor;
- pressure sensors;
- camera (RGBD, external);
- communication (RF sensors);
- speech (microspeaker);
- sound (microphones).

Automated, sensory data-based NTS and workload assessment can be a key to an objective, reproducible approach to measure the surgeon’s skills without bias and the need of human resources. However, these techniques are typically costly, harder to implement and the usage of additional digital tools can be a problem in a clinical environment, even in an Internet of Things setup. Nevertheless, NTS and workload might be demonstrable in objective, technical skills, as suggested in [93], which means these sensors can provide an option for NTS assessment as well. As shown in this article, this research field is not studied widely yet. Automated technology-based studies can be found in Table 5 under the following references: [7,30,34,41,42,43,44,45,46,69,70,72,85,93,111,114,115].

## 4. Discussion

RAMIS related skill assessment is a relatively young research field, and the strong societal need for NTS and workload assessment have not appeared extensively in the literature yet. A few publications suggested objective, sensor-based non-technical skill and mental load evaluation in RAMIS. These approaches can provide a bias-free, reproducible solution in the clinical environment, and allow for the effortless collection of large datasets. Furthermore, during surgical education, personalized skill training would provide a more effective learning procedure, which can be achieved more easily when provided objective metrics. Nevertheless, such metrics are hard to implement, additional sensor usage can always be problematic in the surgical environment, and at the moment, there are no validated objective and automated metrics in NTS assessment. On the other hand, there are close relations in manual MIS and RAMIS, and in manual MIS, it is already suggested to approach NTS assessment with technical skill assessment metrics [38,124], which is a much more deeply studied area in RAMIS. It is assumed that technical and non-technical skills are not different in RAMIS, thus the connections of these two seemingly diverse research approaches shall be studied further. A validated manual technique could be achieved by a relatively simple statistical analysis, but in the case of automated techniques, appropriate test environment, amount of data, sensor usage, feature extraction and classification techniques should all be examined and validated.

For technical skill assessment, there are accurate results with kinematic [125] and video data [126] already. However, these studies only focused on the surgeon and not on the whole staff of the operating room. With external sensors (such as cameras) workflow and NTS (such as communication and teamwork) correlation can be further studied [127]. However, the first step of these studies is to examine the different sensor outputs, which can both correlate with technical and non-technical metrics. RAMIS built-in sensors (3D endoscopic camera and kinematic sensors) can significantly ease NTS and workload assessment, leading to established correlations between sensor outputs and/or self/expert-rating results.

## 5. Conclusions

In this article, we presented the findings of an extensive literature search, performed based on the standard PRISMA method, focusing on the domain of non-technical skill and mental load assessment in Robot-Assisted Minimally Invasive Surgery. Non-technical skills and mental workload directly influence the surgeon’s performance, and thereby the surgical outcome. The importance of non-technical skill assessment in robotic surgery is already recognized, however, there are not too many studies targeting this particular field. In traditional manual MIS, there are already validated metrics for non-technical skill assessment, yet it is clear that robotic surgery requires different non-technical skills from the surgeon, which might be monitored with alternative sensor systems. Alternative skills include advanced teamwork, capabilities to deal with new stress sources and different decisions to make. In the case of RAMIS for mental load assessment, authors often use self-rating techniques, such as NASA-TLX and SURG-TLX, meanwhile, there are no self-rating questionnaires specifically created for RAMIS yet. The existing primary technique for traditional surgery, SURG-TLX, defines the following workload categories: mental demands, physical demands, temporal demands, task complexity, situational stress and distractions. While SURG-TLX is similar to the general NASA-TLX, there are significant differences, such as the examination of distractions in the operating room. It concludes that workload factors for RAMIS can be different as well. For non-technical skill assessment, an often-used technique is expert rating, where a group of expert surgeons assesses the skills of the surgeon based on a validated metric, but this technique can be biased, and may require significant human resources.

The only established expert-rating tool for RAMIS is ICARS, which defines the following non-technical skills for surgeons: communication, teamwork, leadership, decision making, situation awareness and ability to cope with stress and distractions. The final frontier is a sensor-based objective, automated non-technical skill assessment method for RAMIS. Towards this, there are preliminary studies that use physiological signals, such as heart rate or the electrical activity of the brain. Most of the publications examined workload in RAMIS, a significant amount studied brain activity, but specific non-technical skills (in descending order: communication, stress, leadership, decision making, situation awareness and teamwork) can be found in the state-of-the-art as well. At the moment, there exists no widely accepted non-technical skill and mental workload assessment method in the clinical practice of RAMIS.

## Figures and Tables

**Table 1:** NASA-TLX mental workload self-rating questionnaire [18].

Title	Endpoint	Description
Mental demands	low/high	How much mental activity was required?
Physical demands	low/high	How much physical activity was required?
Temporal demands	low/high	How much time pressure did you feel?
Effort	low/high	How hard did you have to work?
Performance	good/poor	How stressful do you think you were?
Frustration level	low/high	How frustrated did you feel?

**Table 2:** SURG-TLX mental workload self-rating questionnaire [87].

Title	Endpoint	Description
Mental demands	low/high	How mentally fatiguing was the procedure?
Physical demands	low/high	How physically fatiguing was the procedure?
Temporal demands	low/high	How hurried or rushed was the pace of the procedure?
Task complexity	low/high	How complex was the procedure?
Situational stress	low/high	How anxious did you feel while performing the procedure?
Distractions	low/high	How distracting was the operating environment?

**Table 3:** Behavioral rating systems in traditional surgery compared to ICARS, the only established non-technical skill assessment metric particularly for RAMIS [21,22].

	Revised NOTECHS	NOTSS	OTAS	ICARS
**Date**	2008	2006	2006	2017
**Reference**	[100]	[101]	[102]	[22]
**Non-technical skills**	Communication and interaction Situational awareness Team skills Leadership and management Decision making	Situational awareness Decision making Task management Leadership Communication Teamwork	Task checklist Shared monitoring Communication Cooperation Coordination Shared leadership	Communication and teamwork Leadership Decision making Situational awareness Cope with stress and distractors
**Content validity**		✓	✓	✓
**Construct validity**			✓	✓
**Inter-rater reliability**	✓	✓	✓	✓
**Sensitivity**	n.a.	not acceptable in some categories	n.a.	n.a.
**Feasibility**	✓ (especially for self-assessment)	✓	limited to certain procedures	✓

**Table 4:** Interpersonal and Cognitive Assessment for Robotic Surgery (ICARS) expert rating metrics [22].

NTS Category	NTS Group	NTS
Interpersonal skills	Communication and teamwork	Effective verbal communication
		Appropriate interaction with bedside surgeon
		Appropriate interaction with operating room staff
		Engages/initiates in confirmatory feedback with OR staff
	Leadership	Appropriate and polite instructions
		Effective workload management
		Coordination of the team from the console
		Coordination of the team at the bedside
		Delegating tasks to team members
		Maintenance of professional standards
Cognitive skills	Decision making	Appropriate decision making in case of equipment failure
		Appropriate decision making at the bedside
		Quick diagnosis of unexpected patient events
		Quick decision making in case of emergency
		Generation, selection and implementation of solutions
		Outcome review of decision
	Situation awareness	Awareness of patient status
		Ability to deal with patient at the bedside
		Ability of quick adaptation to problems
		Anticipation of potential problems
		Role awareness of surrounding team members at the console
Personal resource skills	Cope with stress and distractors	Understands personal limitations and asks for help
		(if necessary)
		Identification of stressor
		Maintenance of cognitive skills
		Maintenance of technical skills
		Professional and appropriate choice of resolution

**Table 5:** Non-technical skill and mental workload assessment in surgical robotics. Used abbreviations: RAMIS: Robot-Assisted Minimally Invasive Surgery, OR: Operating Room, VR: Virtual Reality, EEG: electroencephalogram, NASA-TLX: NASA Task Load Index, SURG-TLX: Surgery Task Load Index, NOTSS: Non-Technical Skills for Surgeons, MRQ: Multiple Resources Questionnaire, DSSQ: Dundee Stress State Questionnaire, ECG: electrocardiogram, HR: heart rate, HRV: heart rate variability, RSME: Rating Scale for Mental Effort, PTICSQ: Psychometric Testing of Interpersonal Communication Skills Questionnaire, SAQ: Safety Attitudes Questionnaire, fNIRS: Functional Near-Infrared Spectroscopy, PVT: Psychomotor Vigilance Test, WCST: Wisconsin Card Sorting Test, CITS: Coping Inventory of Task Stress, MSSD: mean square of successive differences between consecutive heartbeats, PEP: time of isovolumetric contraction, HRA: average heart rate, SMEQ: Subjective Mental Effort Questionnaire, LED: Local Experienced Discomfort, SSSQ: Short Stress State Questionnaire, p.: procedures (where no subject data were available), QoE: Quality of Evidence, mod.: moderate.

Ref.	Date	Subj.	Environment	Input	Measured Feature/NTS	Conclusion	QoE
[30]	2006	10	Dry lab	Skin conductance Self-rating (custom)	Workload Stress	Stress is less in the case of RAMIS compared to traditional MIS.	mod.
[58]	2006	5	VR simulator	NASA-TLX	Workload	Workload can be increased in proportion to delay time with the proposed simulators.	low
[31]	2008	15	Dry lab	DSSQ MRQ CITS	Workload Stress	Stress is less, workload and stress coping strategies are the same in the case of RAMIS compared to traditional MIS.	low
[65]	2009	20	VR simulator	NASA-TLX	Workload	Mimic dV-Trainer shows reasonable workload results.	low
[60]	2009	15	Dry lab	NASA-TLX MRQ	Workload	The usage of the da Vinci 3D view causes less workload compared to the 2D view in some cases.	low
[67]	2009	6	VR simulator	NASA-TLX	Workload	Time delay in teleoperation can significantly increase the workload.	low
[85]	2009	16	Dry lab	MSSD PEP HRA SMEQ LED	Workload Stress	RAMIS causes less cognitive workload compared to traditional MIS.	low
[8]	2010	34	Live porcine	NASA-TLX	Workload	RAMIS poses less mental workload compared to traditional MIS.	mod.
[73]	2010	3	VR simulator	NASA-TLX	Workload	Workload is not improved under delays of 300 ms and 400 ms in the simulated environment.	low
[115]	2010	21	VR simulator	fNIRS	Workload	FNIRS can show the cognitive burden during training.	high
[78]	2012	15	Dry lab	MRQ DSSQ	Workload Stress	Novices have less stress when working with the da Vinci compared to traditional MIS.	low
[74]	2012	12	Dry lab	NASA-TLX	Workload	After the proposed training, mental workload is similar between novices and experts.	low
[114]	2012	21	VR simulator	fNIRS	Cortical activity	There is a significant difference between expert and non-expert subjects with Gaze-Contingent Motor Channeling.	mod.
[7]	2014	2	OR	HR HRV	Stress	RAMIS poses less mental workload compared to traditional MIS. Workload measurement with HRV is cumbersome.	mod.
[55]	2014	28	Dry lab	NASA-TLX	Workload	RAMIS poses significantly better workload perception compared to traditional MIS.	low
[54]	2014	13	Dry lab	NASA-TLX	Workload	Physiological and cognitive ergonomics with robotic surgery are significantly less challenging compared to traditional MIS.	low
[53]	2014	52	VR simulator	NASA-TLX	Workload	Urethrovesical anastomosis VR training improves technical skill acquisition with cognitive demand.	mod.
[93]	2015	10	Dry lab	EEG	Cognitive engagement Mental workload Mental state	Cognitive assessment can define the expertise levels.	high
[42]	2015	32	Dry lab	SURG-TLX RSME Heart rate monitor	Workload HRV	RAMIS poses less mental workload compared to traditional MIS.	mod.
[99]	2015	6	Simulated OR	Expert rating (custom)	Communication Leadership	Repeated simulations and increased leadership mean faster and less flawed conversions in the OR.	mod.
[64]	2015	24	Image display	NASA-TLX	Workload	Increasing the level of cognitive load is significantly increasing the inattention blindness.	mod.
[45]	2015	1	OR	EEG NASA-TLX	Workload Distractions Mental state	Expert surgeons use different mental resources based on their needs.	mod.
[98]	2016	89	OR	Expert rating (custom)	Communication Decision making	RAMIS increases communication requirements for the team of the OR.	mod.
[63]	2016	28	VR simulator	NASA-TLX	Workload	Xperience Team Trainer emphasizes the importance of teamwork.	mod.
[81]	2016	32	OR	PTICSQ SAQ	Communication	There is a significant correlation between team communication and surgical outcome.	mod.
[43]	2016	1	OR	EEG NASA-TLX	Workload	A surgical expert during mentoring concerned while he was observed the surgery.	low
[40]	2016	89	OR	Expert rating (custom) NASA-TLX	Communication Workload	The proposed method is capable of capturing team activities during RAMIS.	mod.
[56]	2016	21	Live porcine VR simulator	NASA-TLX	Workload	Live animal and VR simulator training provide a comparable workload.	low
[70]	2016	8	VR simulator	EEG NASA-TLX	Procedural memory Attention level Workload	EEG can show the learning progress in the case of RAMIS.	high
[59]	2017	55	OR	NASA-TLX	Workload	The study proposes a workload variety analysis with different members of the OR.	mod.
[94]	2017	25 p.	OR	NASA-TLX	Workload	NASA-TLX is a useful tool for determining the appropriate staff member mix for RAMIS procedures.	mod.
[95]	2017	10	OR	SURG-TLX	Workload	Mental demands are higher for surgeons at the console than are assisting.	mod.
[66]	2018	24	Live porcine	NASA-TLX	Workload	Single-site access surgery can significantly reduce the workload.	mod.
[34]	2018	27	VR simulator	EEG NASA-TLX	Cognitive features Mental workload Engagement Asymmetry index Brain functional features Communication Integration Recruitment Workload	EEG features can be used for objective non-technical skill assessment.	high
[61]	2018	27	OR	OR efficiency (custom) NASA-TLX	Communication Workload	Anticipation causes shorter operating time. Team familiarity causes less inconveniences. Less anticipation causes less cognitive load.	mod.
[62]	2018	32	VR simulator	NASA-TLX SSSQ MRQ	Workload Stress	Training with a VR simulator can decrease the workload and stress.	mod.
[33]	2018	62	Dry lab Simulated OR	NOTSS	Situational awareness Decision making Leadership Communication Teamwork	Motor imaginary training technique is not effective in non-technical skill training.	mod.
[44]	2018	8	Dry lab	fNIRS SURG-TLX HRV	Prefrontal activation Workload Stress response	RAMIS improves performance during high workload conditions.	high
[69]	2018	4	OR	EEG NASA-TLX	Cognitive features Functional features Mental workload Mental load Engagement Situation awareness Blink rate Asymmetry index Completion time Communication	During a simple surgical task, functional brain features are sufficient to classify mentor–trainee trust.	high
[111]	2018	32	VR simulator	EEG	Electrocortical activity in temporoparietal and left frontal regions	There are significant differences in electrocortical activity between novices and experts.	high
[72]	2018	12	VR simulator	HRV NASA-TLX Wrist motion EMG Electrodermal EEG	Workload Expertise	The proposed skill and workload evaluation framework is accurate.	high
[32]	2019	20	OR	NOTSS NASA-TLX	Situational awareness Decision making Leadership Communication Teamwork Workload	Non-technical skills are associated with team efficiency, surgical flow disruptions and self-perceived performance.	high
[75]	2019	5	OR	NASA-TLX	Workload	Workload is less in the case of robot-assisted submucosal dissection compared to the traditional case.	low
[68]	2019	31	VR simulator	NASA-TLX	Workload	Specific self-directed robotic simulation curriculum was introduced, which can significantly decrease the workload.	mod.
[41]	2019	8	VR simulator	NASA-TLX Eye movements	Workload	Eye movements correlate with the workload.	high
[71]	2019	264 p.	OR	NASA-TLX	Workload	Mental workload is similar in the case of RAMIS, traditional MIS, hand-assisted MIS and open surgery.	mod.
[57]	2019	30	Wet lab	NASA-TLX PVT WCST	Workload Concentration Cognitive function	Robotic assistance does not provide less mental workload with novices. Robotic assistance may be mentally taxing for robotic novices.	mod.
[76]	2020	7	OR	NASA-TLX	Workload	RAMIS requires less mental demand and effort compared to open access surgery and traditional MIS.	mod.
[46]	2020	26	Dry lab	Task-evoked pupillary response	Workload	Under high cognitive workload, there can be a divergence in robotic movement profiles between expertise levels.	high
[96]	2020	n.a.	OR	OTAS NOTSS ICARS NOTECHS II	Situation awareness Decision making Communication Teamwork Leadership Stress	The study proposed a structured approach to the analysis of non-technical skill using extracorporeal videos of both open radical cystectomy and RAMIS radical cystectomy	mod.
